# Gender balance in Australian pharmacy organisations: Are we there yet?

**DOI:** 10.1016/j.rcsop.2024.100442

**Published:** 2024-04-12

**Authors:** Thao Linh Pham, Gregory M. Peterson, Alicia Martin, Mark Naunton

**Affiliations:** a Discipline of Pharmacy, Faculty of Health, University of Canberra, Bruce, Canberra, ACT 2617, Australia; b School of Pharmacy and Pharmacology, University of Tasmania, Hobart, TAS 7001, Australia; c Community Pharmacist, Canberra, ACT, Australia

**Keywords:** Equality, Gender, Leadership, Pharmacy, Gender balance, Gender equity

## Abstract

**Introduction:**

In the dynamic landscape of healthcare, pharmacists play a critical role in ensuring the well-being of communities, and having solid professional organisations to support pharmacists is essential in crucial activities, including continuing education, advocacy and establishing service standards. Eight pharmacy organisations play vital roles in representing pharmacists in various sectors and collectively contribute to developing, regulating, and promoting the pharmacy profession in Australia. However, a notable lack of female representation in these organisations' leadership roles has led to an increased focus on gender balance and equity.

**Objective:**

To determine if the gender distribution in pharmacy leadership aligns with the pharmacy workforce in Australia (64% women) and how it has changed in the five years since our last study on the issue.

**Setting:**

Australia.

**Method:**

Eight key Australian pharmacy organisations were identified. The website for each organisation was accessed, and data were recorded for their 2023 boards/committees/councils based on annual reports. Data recorded include name, number of males, number of females, and the gender of the president/chair of each board/committee/council.

**Results:**

Data were obtained for 340 separate professional committee members from the eight organisations (including state/territory branches) in 2023. Gender balance in pharmacy organisations has increased significantly since 2018, with women's representation in leadership positions now at 58% (47% 2018).

**Conclusion:**

Gender equity within Australian pharmacy professional organisations has significantly progressed.

## 1 Introduction

In the dynamic landscape of healthcare, pharmacists play a critical role in ensuring the well-being of communities.^1^^,^^2^ However, while the profession has made significant strides in recent years, it still grapples with gender imbalance and equity issues within its leadership ranks.^3^, ^4^, ^5^ The subject of gender balance and equity in leadership in pharmacy has gained increasing attention, reflecting a broader global discourse on the need for diversity and inclusion in all sectors.^5^^,^^6^

A study of gender equity in pharmacy academia found that women occupy fewer positions of power and leadership in the profession and are less represented on editorial boards.^3^ From 2015 to 2020, only about 23% of chief executive officer (CEO) deans in pharmacy academia were women in the US.^3^^,^^7^ Moreover, a report by Global Health 50/50 showed that men lead 69% of global health organisations, and 80% of board chairs in global health were men.^8^ Various factors may be implicated, including gender biases, stereotypes, and workplace cultures, that may not provide equal opportunities for women to advance to senior leadership positions.^9^^,^^10^ Despite qualifications and achievements, women may encounter biases in the hiring and promotion processes.^10^ Stereotypes, such as effective leadership, require assertiveness and decisiveness. These traits are often associated with masculinity, leading to a negative perception of women’s ability to take on leadership roles. Women leaders may be perceived as too collaborative or nurturing, rather than authoritative ^11^^,^^12^

There is concern that gender disparity in healthcare leadership will lead to a depletion of crucial skills and experience, heightened workforce sustainability costs, and negative repercussions on healthcare and policies that specifically impact women and children.^13^^,^^14^ The underrepresentation of women in pharmacy leadership roles also raises ethical concerns regarding fairness and equity, limits role modelling and mentorship opportunities, and hampers workforce diversity and innovation, which could result in economic consequences such as lost talent and decreased productivity.^13^^,^^14^ For example, studies looking at women nurses in healthcare leadership found that this lack of representation can result in healthcare interventions that are less effective or appropriate for women, contributing to disparities in healthcare outcomes.^15^ Therefore, promoting and achieving gender equality in pharmacy leadership has significant implications for the profession and the healthcare system.^10^^,^^16^ Gender-diverse leadership in pharmacy can better reflect the diverse needs of the workforce and the patient population, leading to improved performance and overall organisational success.^10^^,^^16^ Gender equality ensures diverse perspectives, ideas, and approaches within pharmacy leadership.^10^^,^^15^ This diversity can foster innovation and creative problem-solving, ultimately benefiting the development of pharmaceutical practices, policies, and strategies.^15^

A balanced representation of genders in leadership roles is crucial to lead to a more innovative, inclusive, and efficient healthcare system.^10^ Ensuring equal opportunities for both genders in leadership positions can contribute to a meritocracy where individuals are selected based on qualifications and competencies rather than gender, resulting in a more skilled and capable leadership team.^17^^,^^18^ Moreover, gender equality promotes a more inclusive and supportive work environment, increasing job satisfaction, motivation, and overall well-being among pharmacy professionals, leading to higher levels of productivity and efficiency.^17^^,^^18^

The significance of diversity in leadership within healthcare cannot be overstated. Gender equality in leadership roles brings diverse perspectives, experiences, and insights to decision-making processes, leading to more innovative solutions, improved patient care, and a more robust organisational culture.^18^^,^^19^ Selecting individuals based on their qualifications, skills, and merits rather than solely on their gender is essential for fostering a fair and effective healthcare system.^17^ Selecting candidates solely based on gender, even with positive intentions, risks reinforcing stereotypes, unintentionally prolongs a distinct type of prejudice and hinders the advancement of genuine inclusivity.^20^ Utilising that meritocratic approach will ensure that the best-suited individuals are appointed to positions, maximising the quality of care provided and promoting a fair and effective working environment.^17^

A wealth of literature discusses gender inequality and its effects on leadership in various fields outside of pharmacy.^21^ The pharmacy profession could use this information to formulate an evidence-based position.^21^ Our previous study, conducted in 2018, explored gender balance in pharmacy leadership in Australia and how it has changed over the last 20 years.^5^ It showed that while there was still a presence of gender disparities in Australian pharmacy organisations, it had improved over time, and a target of 50/50 gender equity was achievable by 2030.^5^ Five years later, whether the pharmacy profession is still on track to meet that target remains. Therefore, this project aimed to assess if the gender composition of leadership positions in Australian pharmacy organisations aligns with the gender distribution within the pharmacy workforce since 2018. By completing this study, we hoped to determine whether we are on track to address gender inequality and further contribute to promoting diversity and inclusion in pharmacy leadership roles.

It is important to note that gender refers to how a person identifies or expresses their masculine or feminine characteristics, and determining this requires discussion with the individual. In this research, we examined “assumed” gender in a simple binary manner based on recorded names, photographs, and biographical descriptions.

### 1.1 Ethics approval

Ethics approval was not required as this research was based on publicly available data.

## 2 Methods

We replicated the approach of our previous study.^5^ Eight key national Australian pharmacy organisations were identified: the Pharmaceutical Society of Australia (PSA), the Pharmacy Guild of Australia (PGA), the Society of Hospital Pharmacists of Australia (SHPA), Pharmaceutical Defence Limited (PDL), the Pharmacy Board of Australia (PBA), Professional Pharmacists Australia (PPA), the Australian Pharmacy Council (APC) and the National Australian Pharmacy Students' Association (NAPSA). More details of each organisation are included in our previous study.^5^ The PSA, PGA, PDL, and SHPA have individual state/territory branches, also included in the study. Leadership positions were determined as playing significant roles in governing and managing organisations, such as president, board member, or committee chair. Therefore, the website for each organisation was accessed by one researcher (AP), and data were recorded for the 2023 boards/committees/councils based on annual reports. Data recorded were names, biographical details (when listed), the number of (assumed) men and women, and the gender of each organisation's chair and committee member. We also accessed publicly available profiles and pictures from LinkedIn or public articles. We conducted a 20% random check of the data with another researcher (MN) to ensure data reliability. The percentage of males and females from each organisation was then calculated. Data collection ran from August to October 2023. Pharmacy workforce statistics in Australia for 2023 were obtained from the statistics section of the PBA website.^19^

## 3 Results

Information was collected from all organisations, and data were obtained for 340 professional committee members from the eight organisations (including state/territory branches from the PSA, PGA, PDL, and SHPA). There was a rise in the proportion of women – to 58% in 2023, compared with 47% in 2018. Only two organisations (SHPA and NAPSA) exceeded 50% of female leadership representation in 2018; in contrast, six out of the eight organisations achieved this in 2023.

**Fig. 1:**
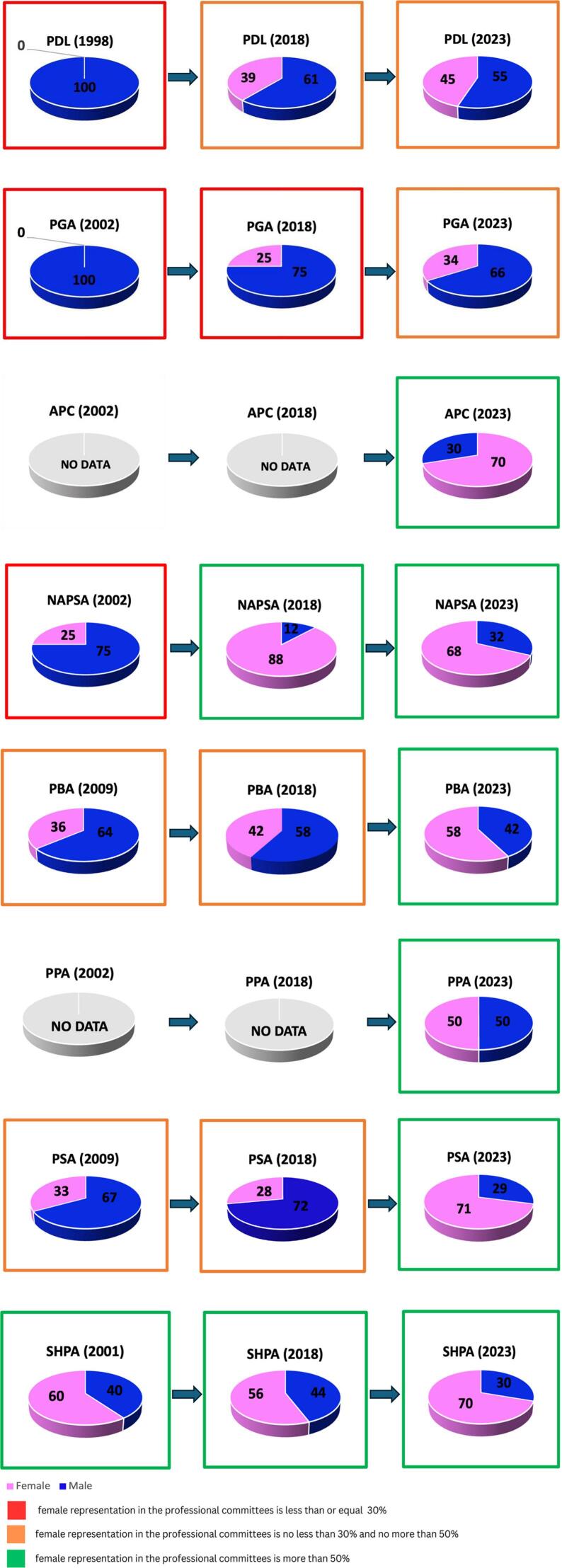
Percentage of males/females on national professional committees from past year data to present.

Over 70% of the APC, PSA, and SHPA committee members were female. Fig. 1 summarises gender balance on the national professional committees of each organisation between 1998 and 2023. We used a traffic light system to colour-code the progress being made. We defined *poor gender balance* as less than or equal to 30% of females with a red box.^20^ Every organisation had at least 30% female leadership representation in 2023, so no red box is presented.

## 4 Discussion

Gender inequality in pharmacy leadership has been a concern due to its impact on limiting diverse perspectives in decision-making processes. It undermines the profession's ability to address the diverse needs of patients and society.^4^^,^^5^^,^^22^ This underrepresentation can perpetuate gender biases within the healthcare system.^15^ However, we can see that the proportion of female pharmacists in Australia has steadily increased over the past few decades. The latest statistics show that in 2023, 64% of Australian pharmacists were female, and 36% were male.^22^ The critical pharmacy professional organisations must reflect this distribution within their leadership ranks. Data were obtained for 340 professional committee members from 8 organisations, including all Australian state/ territory branches, in 2023. There has been a noteworthy shift in the gender composition of professional leadership committees within pharmacy. By 2023, female representation had significantly increased, constituting 58% of committee members.

There has been significant progress in gender balance in almost all Australian pharmacy organisations since our previous study.^5^ Women now occupy more leadership positions in the profession and are relatively well-represented. The progress made contrasts with the negative observations in the systematic review by Mousa et al., “The healthcare sector, with a primarily female workforce, is currently advancing women in leadership at a glacial pace” and “The barriers to advancing women in healthcare leadership are well entrenched and understood, the case for change is compelling, yet the challenges seem intractable, and progress has been slow”.^23^

PSA, SPHA, and APC have a notable female leadership representation of around 70%. Despite improving, PGA and PDL still have not achieved equal gender distribution; only 34% and 45% of the professional committee members for PGA and PDL, respectively, were women in 2023. This finding probably reflects the gender distribution of pharmacy ownership in Australia.

This observed increase in female leadership representation within the pharmacy sector fosters a more diverse and inclusive organisational culture.^8^^,^^9^ Female leaders can bring diverse viewpoints and experiences to decision-making, leading to more comprehensive and effective outcomes. Pharmacy organisations, therefore, are better equipped to address complex challenges, innovate, and adapt to changing industry dynamics.^8^^,^^9^^,^^24^ The effort and contribution from all the organisations must be acknowledged as we have seen progress in achieving gender equality in all organisations. For example, PSA has started a pharmacy leadership program called Ignite. This program began in 2019 and aims to empower high-performing early-career pharmacists into future leaders, contributing to the development from 2018 to the present.

Regarding strategies to promote gender equality, it is crucial to pinpoint and tackle diverse obstacles rather than solely relying on quotas. More impactful approaches involve implementing policies that ensure equal opportunities for men and women in hiring, promotion, and leadership positions within pharmacy settings.^25^ Flexible work arrangements such as part-time options, job-sharing, or remote work are offered to accommodate the needs of both male and female pharmacists, particularly those with family responsibilities.^25^^,^^26^ Training programs and workshops focused on gender bias awareness, leadership skills development, and work-life balance strategies to empower both male and female pharmacists to thrive.^25^ Most importantly, it addresses gender stereotypes within the pharmacy field, fostering an environment where all pharmacists are recognised and valued for their skills, knowledge, and contributions, regardless of gender.^25^, ^26^, 27. By implementing these strategies, pharmacy organisations can work toward creating a more equitable and inclusive environment that promotes gender balance and empowers all pharmacists to succeed.

We acknowledge that having a position on a professional committee does not automatically confer leadership abilities. This study focuses solely on committee positions and overlooks numerous other realms of leadership in the pharmacy field, such as ownership and management roles spanning various sectors like community pharmacy, hospitals, academia, and government. Further investigation into gender distributions in these additional leadership positions is necessary to understand the gender balance in pharmacy leadership in Australia. Moreover, we propose introducing training initiatives focused on unconscious bias to heighten individual awareness regarding any unintentional biases they might harbour. We also acknowledge that it was unknown, based on the available lists, whether anyone was identified as non-binary in terms of gender. We highlight this limitation and acknowledge that we could not definitively categorise or identify non-binary individuals.

While the data addresses the primary goal of evaluating gender balance in pharmacy leadership, it is crucial to highlight the limitation of exclusively concentrating on gender distribution. Future studies should explore additional metrics such as performance indicators, leadership effectiveness, and competency exams to gain a more thorough insight. Such an approach would enhance the ongoing gender equality assessment in pharmacy organisations. Furthermore, future studies about signs of gender oppression or discrimination within the pharmacy profession should also be conducted to help unveil potential discrepancies and guide efforts toward fostering equitable career pathways. It is also important to emphasise the importance and acknowledge the contribution of male leadership within the pharmacy profession. It is essential to understand both sides of the gender equation.

Leadership development requirements apply to individuals of all genders. However, a common belief is that proficient women in the pharmacy workforce require support to actively seek leadership roles and overcome obstacles specific to women.^8^^,^^28^ These barriers could stem from societal gender role expectations or perceived differences in skills and leadership strengths based on gender.^8^ Motivating women to pursue leadership positions not only expands the talent pool for the pharmacy profession but also addresses a crucial necessity for current and future leadership.^8^ We are on track to reach a target of 50/50 and it is expected to be sooner than 2030.

## 5 Conclusion

Acknowledging that we could not definitively categorise or identify non-binary individuals, gender equity within the leadership of Australian pharmacy professional organisations has made significant progress, reflecting the growing recognition of the valuable contributions made by women pharmacists. This achievement is essential to fostering inclusivity and gender parity within pharmacy and promoting a more representative healthcare landscape. Future research should examine the representation within leadership roles of other gender identities and minority groups within the pharmacy profession.

## Funding

No funding was received for this project.

## CRediT authorship contribution statement

**Thao Linh Pham:** Writing – original draft, Methodology, Investigation, Formal analysis, Data curation, Conceptualization. **Gregory M. Peterson:** Writing – review & editing. **Alicia Martin:** Writing – review & editing. **Mark Naunton:** Writing – review & editing, Supervision, Resources, Project administration, Methodology, Conceptualization.

## Declaration of competing interest

None
